# Supplementary material for: Identifying Genetic Variants and Metabolites Associated with Rapid Estimated Glomerular Filtration Rate Decline in Korea Based on Genome–Metabolomic Integrative Analysis
Source: Metabolites. 2022 Nov 19;12(11):1139. doi: 10.3390/metabo12111139 (PMC9695695; doi:10.3390/metabo12111139)
Supplement: Supplementary file 1 [file metabolites-12-01139-s001.zip › metabolites-2022380-supplementary.pdf]

Supplementary Material Table of Contents

1) Figure S1. Study population and workflow for the genome-metabolomics integrative analysis (GMIA).
2) Figure S2. Manhattan and quantile-quantile plots of GWAS for the rapid decline in the estimated glomerular filtration rate. (A) K-CHIP consortium: discovery dataset. (B) KNOW-CKD cohort: validation dataset. GWAS, genome-wide association study; K-CHIP, Korean Chip; KNOW-CKD, The KoreaN Cohort Study for Outcomes in Patients With Chronic Kidney Disease.
3) Figure S3. Associations between SNPs and the serum creatine concentration using a linear regression model from GMIA. (A) G: reference allele; C: effect allele of rs183537972 (HYAL6P). (B) C: reference allele; T: effect allele of rs145083655 (HYAL4). (C) G: reference allele; A: effect allele of rs116869578 (LMOD2). (D) G: reference allele; A: effect allele of rs148528467 (WASL). GMIA, genome-metabolomics integrative analysis; SNP, single nucleotide polymorphism.
4) Table S1. List of 135 serum metabolites in the KARE cohort
5) Table S2. Genome-wide association study-derived genetic variants in the K-CHIP consortium
6) Table S3. Fine-mapping of the lead SNPs from the GWAS results

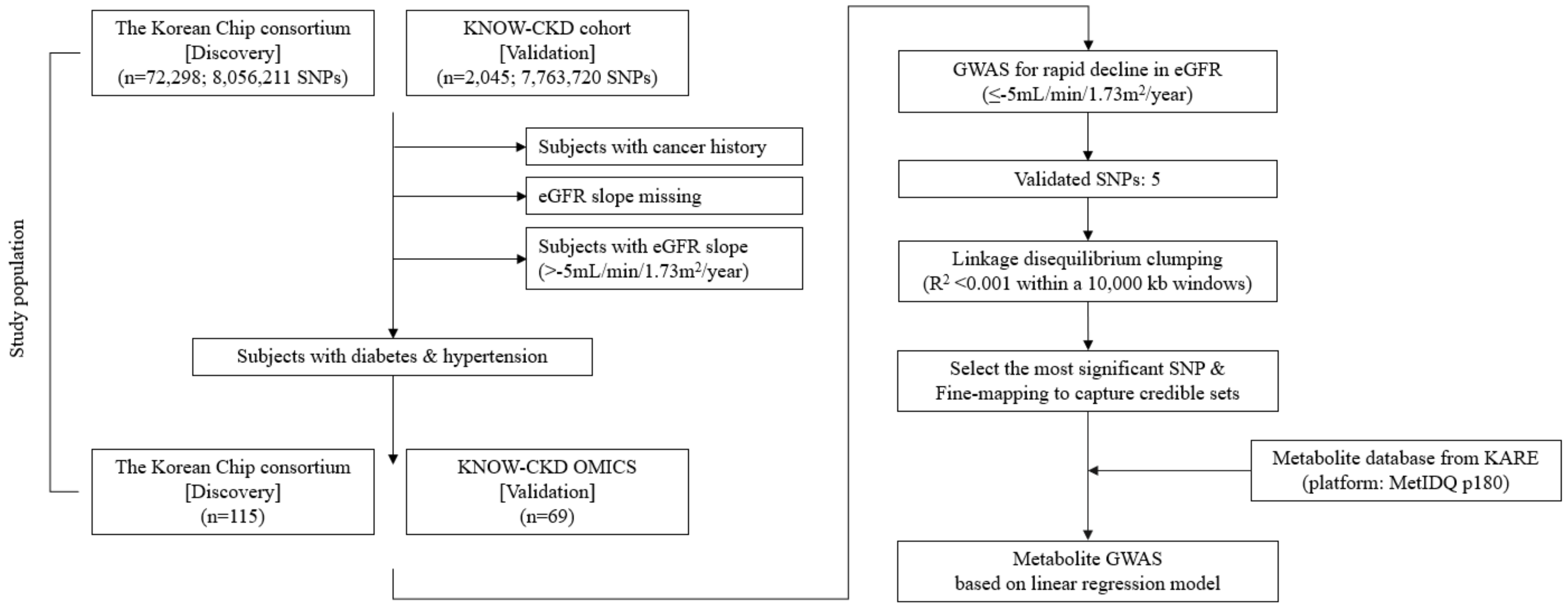

Figure S1. Study population and workflow for the genome-metabolomics integrative analysis (GMIA).

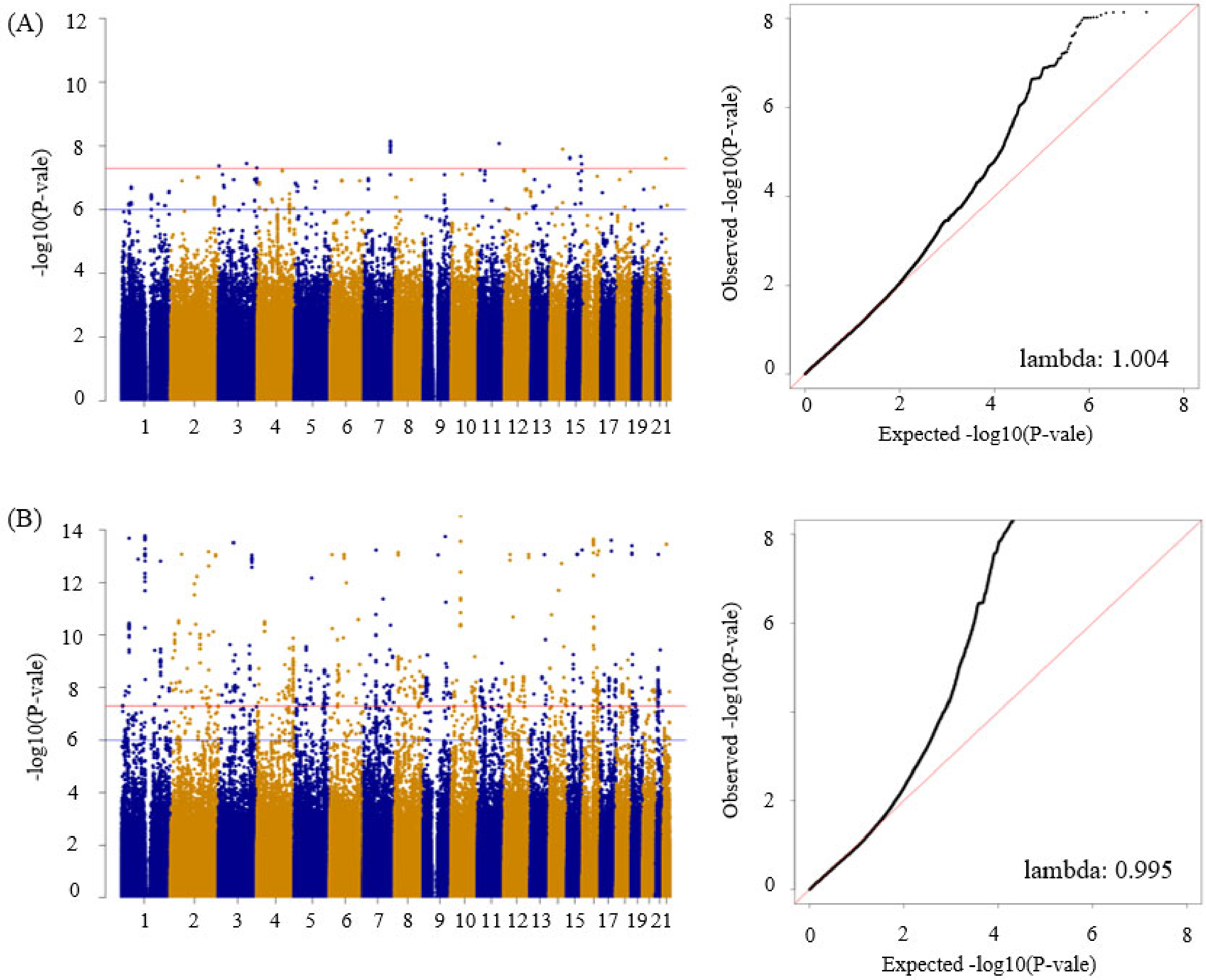

Figure S2. Manhattan and quantile-quantile plots of GWAS for the rapid decline in the estimated glomerular filtration rate. (A) K-CHIP consortium: discovery dataset. (B) KNOW-CKD cohort: validation dataset. GWAS, genome-wide association study; K-CHIP, Korean Chip; KNOW-CKD, The KoreaN Cohort Study for Outcomes in Patients With Chronic Kidney Disease.

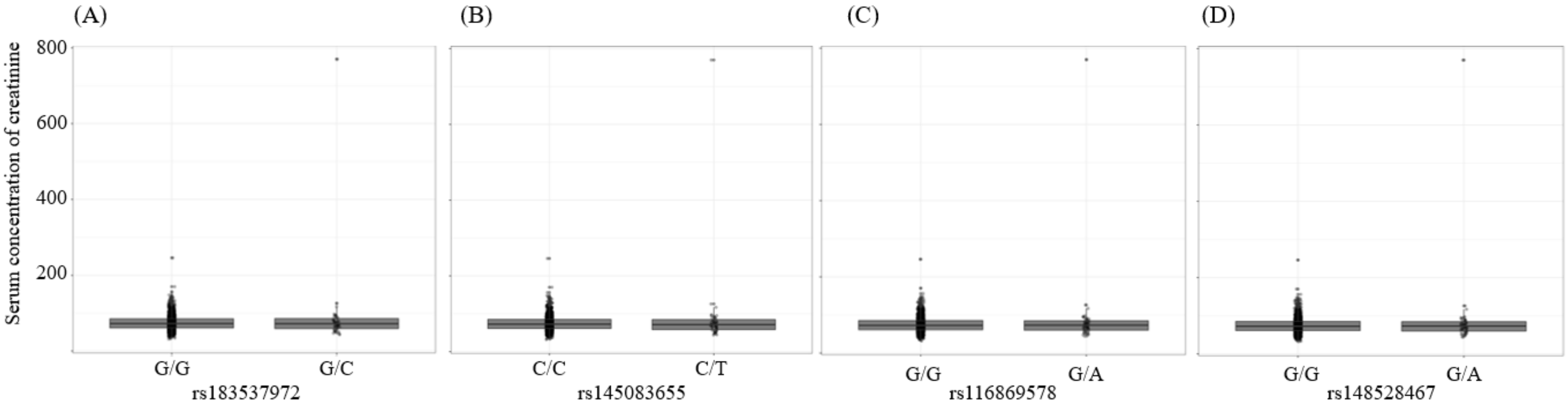

Figure S3. Associations between SNPs and the serum creatine concentration using a linear regression model from GMIA. (A) G: reference allele; C: effect allele of rs183537972 (HYAL6P). (B) C: reference allele; T: effect allele of rs145083655 (HYAL4). (C) G: reference allele; A: effect allele of rs116869578 (LMOD2). (D) G: reference allele; A: effect allele of rs148528467 (WASL). GMIA, genome-metabolomics integrative analysis; SNP, single nucleotide polymorphism.

Table S1. List of 135 serum metabolites in the KARE cohort

Acylcarnitine	Amino acids	Biogenic amines	Sugar	Lyso Phosphatidylcholine	Phosphatidylcholine diacyl
Carnitine	Alanine	Acetylornithine	Hexose	Lyso Phosphatidylcholine acyl C16:0	Phosphatidylcholine diacyl C28:1
Acetylcarnitine	Arginine	Asymmetric dimethylarginine		Lyso Phosphatidylcholine acyl C16:1	Phosphatidylcholine diacyl C30:0
Propionylcarinitine	Asparagine	Creatinine		Lyso Phosphatidylcholine acyl C17:0	Phosphatidylcholine diacyl C32:0
Butyrylcarnitine	Aspartate	Kynurenine		Lyso Phosphatidylcholine acyl C18:0	Phosphatidylcholine diacyl C32:1
Valerylcarnitine	Citrulline	Putrescine		Lyso Phosphatidylcholine acyl C18:1	Phosphatidylcholine diacyl C32:3
Pimelylcarnitine	Glutamine	Sarcosine		Lyso Phosphatidylcholine acyl C18:2	Phosphatidylcholine diacyl C34:1
Octanoylcarnitine	Glutamate	Serotonin		Lyso Phosphatidylcholine acyl C20:3	Phosphatidylcholine diacyl C34:2
Tetradecenoylcarnitine	Glycine	Spermidine		Lyso Phosphatidylcholine acyl C20:4	Phosphatidylcholine diacyl C34:3
Tetradecadienylcarnitine	Histidine	Spermine			Phosphatidylcholine diacyl C34:4
Hexadecanoylcarnitine	Isoleucine	Taurine			Phosphatidylcholine diacyl C36:0
Ocatdecanoylcarnitine	Leucine				Phosphatidylcholine diacyl C36:1
Octadecenoylcarnitine	Lysine				Phosphatidylcholine diacyl C36:2
Octadecadienylcarnitine	Methionine				Phosphatidylcholine diacyl C36:3
	Ornithine				Phosphatidylcholine diacyl C36:4
	Phenylalanine				Phosphatidylcholine diacyl C36:5
	Proline				Phosphatidylcholine diacyl C36:6
	Serine				Phosphatidylcholine diacyl C38:0
	Threonine				Phosphatidylcholine diacyl C38:1
	Tryptophan				Phosphatidylcholine diacyl C38:3
	Tyrosine				Phosphatidylcholine diacyl C38:4
	Valine				Phosphatidylcholine diacyl C38:5
					Phosphatidylcholine diacyl C38:6
					Phosphatidylcholine diacyl C40:1
					Phosphatidylcholine diacyl C40:2
					Phosphatidylcholine diacyl C40:3
					Phosphatidylcholine diacyl C40:4
					Phosphatidylcholine diacyl C40:5
					Phosphatidylcholine diacyl C40:6
					Phosphatidylcholine diacyl C42:0
					Phosphatidylcholine diacyl C42:1
					Phosphatidylcholine diacyl C42:2
					Phosphatidylcholine diacyl C42:4
					Phosphatidylcholine diacyl C42:5
					Phosphatidylcholine diacyl C42:6

KARE, the Korea Association Resource

Table S1. List of 135 serum metabolites in the KARE cohort (Continued)

Phosphatidylcholine acyl-alkyl	Sphingolipids
Phosphatidylcholine acyl-alkyl C30:0	Hydroxysphingomyelin C14:1
Phosphatidylcholine acyl-alkyl C32:1	Hydroxysphingomyelin C16:1
Phosphatidylcholine acyl-alkyl C32:2	Hydroxysphingomyelin C22:1
Phosphatidylcholine acyl-alkyl C34:0	Hydroxysphingomyelin C22:2
Phosphatidylcholine acyl-alkyl C34:1	Hydroxysphingomyelin C24:1
Phosphatidylcholine acyl-alkyl C34:2	Sphingomyelin C16:0
Phosphatidylcholine acyl-alkyl C34:3	Sphingomyelin C16:1
Phosphatidylcholine acyl-alkyl C36:0	Sphingomyelin C18:0
Phosphatidylcholine acyl-alkyl C36:1	Sphingomyelin C18:1
Phosphatidylcholine acyl-alkyl C36:2	Sphingomyelin C24:0
Phosphatidylcholine acyl-alkyl C36:3	Sphingomyelin C24:1
Phosphatidylcholine acyl-alkyl C36:4	Sphingomyelin C26:1
Phosphatidylcholine acyl-alkyl C36:5	
Phosphatidylcholine acyl-alkyl C38:0	
Phosphatidylcholine acyl-alkyl C38:1	
Phosphatidylcholine acyl-alkyl C38:2	
Phosphatidylcholine acyl-alkyl C38:3	
Phosphatidylcholine acyl-alkyl C38:4	
Phosphatidylcholine acyl-alkyl C38:5	
Phosphatidylcholine acyl-alkyl C38:6	
Phosphatidylcholine acyl-alkyl C40:1	
Phosphatidylcholine acyl-alkyl C40:2	
Phosphatidylcholine acyl-alkyl C40:3	
Phosphatidylcholine acyl-alkyl C40:4	
Phosphatidylcholine acyl-alkyl C40:5	
Phosphatidylcholine acyl-alkyl C40:6	
Phosphatidylcholine acyl-alkyl C42:0	
Phosphatidylcholine acyl-alkyl C42:1	
Phosphatidylcholine acyl-alkyl C42:2	
Phosphatidylcholine acyl-alkyl C42:3	
Phosphatidylcholine acyl-alkyl C42:4	
Phosphatidylcholine acyl-alkyl C42:5	
Phosphatidylcholine acyl-alkyl C44:3	
Phosphatidylcholine acyl-alkyl C44:4	
Phosphatidylcholine acyl-alkyl C44:5	
Phosphatidylcholine acyl-alkyl C44:6	

KARE, the Korea Association Resource

Table S2. Genome-wide association study-derived genetic variants in the K-CHIP consortium

Chr	BP	SNP	Function	Gene	Alleles	MAF	Beta (SE)	P-value
7q33	135579485	rs12674250	intergenic	FAM180A;LUZP6	C/T	0.014	-6.632 (1.060)	7.20E-09
7q33	135578048	rs71539472	intergenic	FAM180A;LUZP6	G/C	0.014	-6.630 (1.060)	7.26E-09
7q33	135578725	rs11773428	intergenic	FAM180A;LUZP6	G/T	0.014	-6.631 (1.060)	7.28E-09
7q33	135572294	rs12670070	intergenic	FAM180A;LUZP6	C/T	0.014	-6.622 (1.060)	7.54E-09
11q22.3	105427302	rs117713934	intergenic	CARD18;GRIA4	T/G	0.035	-4.126 (0.663)	8.40E-09
7q33	135567068	rs2348907	intergenic	FAM180A;LUZP6	A/C	0.013	-6.576 (1.060)	9.43E-09
7q33	135570157	rs11762987	intergenic	FAM180A;LUZP6	A/G	0.013	-6.577 (1.061)	9.48E-09
7q33	135527463	rs71539459	intergenic	FAM180A;LUZP6	A/C	0.013	-6.562 (1.059)	9.65E-09
7q33	135531147	rs71539462	intergenic	FAM180A;LUZP6	T/C	0.013	-6.562 (1.059)	9.69E-09
7q33	135551217	rs79466054	intergenic	FAM180A;LUZP6	C/T	0.013	-6.562 (1.059)	9.71E-09
7q33	135537495	rs11975634	intergenic	FAM180A;LUZP6	A/G	0.013	-6.561 (1.059)	9.75E-09
7q33	135520471	rs56244135	intergenic	FAM180A;LUZP6	T/C	0.013	-6.542 (1.060)	1.10E-08
14q31.1	83299627	rs117363978	intergenic	LINC02301;NONE	G/A	0.029	-5.293 (0.862)	1.27E-08
7q33	135490878	rs11766426	intergenic	FAM180A;LUZP6	C/G	0.014	-6.504 (1.063)	1.38E-08
7q33	135539970	rs60112348	intergenic	FAM180A;LUZP6	A/G	0.012	-6.789 (1.113)	1.52E-08
15q26.1	89195127	rs111437463	UTR5	ISG20	G/A	0.008	-9.620 (1.595)	2.08E-08
15q13.3	33261838	rs7168080	intronic	FMN1	T/C	0.027	-4.511 (0.750)	2.26E-08
22q12.2	30757436	rs79247733	intronic	CCDC157	G/A	0.087	-2.734 (0.456)	2.44E-08
15q13.3	33262276	rs60847748	intronic	FMN1	G/C	0.028	-4.487 (0.748)	2.46E-08
3q24	143157268	rs13093275	intronic	SLC9A9	G/T	0.113	-2.304 (0.389)	3.53E-08
15q26.1	93906143	rs148163587	intergenic	LOC105370980;LINC02207	C/T	0.013	-6.334 (1.071)	3.65E-08
3p26.2	3175601	rs149885665	intronic	TRNT1	C/T	0.010	-7.688 (1.307)	4.19E-08

Chr, chromosome; SNP, single nucleotide polymorphisms; MAF, minor allele frequency; SE, standard error; K-CHIP, the Korea Biobank Array

Table S2. Genome-wide association study-derived genetic variants in the K-CHIP consortium (Continued)

Chr	BP	SNP	Function	Gene	Alleles	MAF	Beta (SE)	P-value
3q29	195847118	rs145588336	intergenic	TFRC;LINC00885	C/T	0.048	-3.406 (0.582)	4.85E-08
11p15.4	9650309	rs117764660	intergenic	WEE1;SWAP70	A/G	0.009	-7.626 (1.311)	5.72E-08
12q23.1	98213754	rs139661669	intergenic	LOC643711;MIR4495	T/G	0.009	-7.626 (1.311)	5.72E-08
4q28.1	127056018	rs149461767	intergenic	MIR2054;INTU	C/G	0.013	-7.011 (1.206)	5.75E-08
15q26.1	93888877	rs955690599	intergenic	LOC105370980;LINC02207	C/T	0.014	-6.316 (1.089)	6.08E-08
11p13	34728253	rs184451962	intergenic	EHF;APIP	G/A	0.000	-541.650 (93.367)	6.10E-08
4q28.1	127191674	rs75153312	intergenic	MIR2054;INTU	T/A	0.011	-7.555 (1.303)	6.14E-08
12q23.1	98211000	rs79833465	intergenic	LOC643711;MIR4495	T/C	0.009	-7.612 (1.313)	6.15E-08
18q22.2	67318159	rs73463973	intronic	DOK6	A/G	0.115	-2.110 (0.364)	6.38E-08
15q24.3	77336133	rs118012826	UTR3	TSPAN3	G/A	0.027	-4.485 (0.779)	7.38E-08
10q21.1	53171297	rs117744777	intronic	PRKG1	C/T	0.000	-7739.970 (1346.350)	7.76E-08
11p13	34737478	rs187796610	intergenic	EHF;APIP	A/G	0.000	-540.784 (94.068)	7.76E-08
3p24.3	21653781	rs147909135	intronic	ZNF385D	G/A	0.009	-7.555 (1.315)	7.90E-08
7q33	135549647	rs11764776	intergenic	FAM180A;LUZP6	C/A	0.009	-7.558 (1.316)	7.91E-08
9q31.1	104899256	rs150890582	intergenic	GRIN3A;LINC00587	G/A	0.009	-7.553 (1.315)	7.96E-08
16q22.1	69186529	rs557474024	intronic	UTP4	C/T	0.008	-8.489 (1.485)	8.97E-08
2q22.1	138442200	rs549533033	intergenic	THSD7B;LOC101928273	G/A	0.008	-8.487 (1.489)	9.68E-08
2q22.1	138442195	rs567460414	intergenic	THSD7B;LOC101928273	A/G	0.008	-8.487 (1.489)	9.68E-08
7p15.3	21587183	rs201198710	intronic	DNAH11	ATAAT/A	0.011	-7.470 (1.315)	1.06E-07
3q29	195809725	rs17091661	upstream	TFRC	C/G	0.052	-3.234 (0.569)	1.07E-07
13q13.3	36255134	rs142727278	intergenic	NBEA;LINC00445	C/T	0.017	-5.902 (1.042)	1.15E-07
3q29	192624876	rs116204719	intronic	MB21D2	A/T	0.009	-7.474 (1.320)	1.15E-07

Chr, chromosome; SNP, single nucleotide polymorphisms; MAF, minor allele frequency; SE, standard error; K-CHIP, the Korea Biobank Array

Table S2. Genome-wide association study-derived genetic variants in the K-CHIP consortium (Continued)

Chr	BP	SNP	Function	Gene	Alleles	MAF	Beta (SE)	P-value
3p12.3	77523031	rs145860157	intronic	ROBO2	A/C	0.009	-7.469 (1.319)	1.15E-07
8q21.13	81610194	rs148850681	intronic	ZNF704	T/C	0.009	-7.469 (1.319)	1.15E-07
18p11.21	13719612	rs184493441	intronic	FAM210A	T/C	0.009	-7.469 (1.319)	1.15E-07
3q27.2	185140359	rs76390866	intronic	MAP3K13	C/T	0.009	-7.469 (1.319)	1.15E-07
3q29	192617374	rs141743783	intronic	MB21D2	A/G	0.009	-7.472 (1.320)	1.16E-07
3q29	192621063	rs79926231	intronic	MB21D2	G/T	0.009	-7.472 (1.320)	1.16E-07
3q29	192623144	rs115525172	intronic	MB21D2	G/A	0.009	-7.471 (1.320)	1.16E-07
3q29	192627068	rs11921144	intronic	MB21D2	A/T	0.009	-7.471 (1.320)	1.16E-07
3q29	192626691	rs79654963	intronic	MB21D2	A/T	0.009	-7.471 (1.320)	1.16E-07
3q29	192614938	rs138060225	intronic	MB21D2	A/C	0.009	-7.468 (1.319)	1.17E-07
3q29	192621921	rs77225906	intronic	MB21D2	G/C	0.009	-7.470 (1.320)	1.17E-07
3q29	192630923	rs73198800	intronic	MB21D2	T/C	0.009	-7.464 (1.320)	1.19E-07
6p11.2	57281388	rs575341105	intronic	PRIM2	G/C	0.009	-7.712 (1.364)	1.20E-07
7p15.3	21580775	rs115451224	intergenic	SP4;DNAH11	G/T	0.009	-7.463 (1.321)	1.21E-07
11p13	32360746	rs55767723	intergenic	SNORA88;WT1	C/T	0.012	-7.120 (1.260)	1.22E-07
7p15.3	21583910	rs56688731	intronic	DNAH11	T/G	0.009	-7.458 (1.321)	1.24E-07
6q25.1	150329057	rs185234953	intergenic	RAET1K;RAET1L	A/G	0.024	-4.892 (0.867)	1.25E-07
2p16.1	57600654	rs147118782	intergenic	CCDC85A;VRK2	T/C	0.010	-8.573 (1.519)	1.25E-07
6p11.2	57324187	rs1192133075	intronic	PRIM2	A/C	0.009	-7.919 (1.404)	1.26E-07
6p11.2	57325078	rs994418306	intronic	PRIM2	G/A	0.009	-7.922 (1.404)	1.26E-07
6p11.2	57326277	rs1179007536	intronic	PRIM2	C/T	0.009	-7.926 (1.405)	1.27E-07
6p11.2	57325945	rs1397294397	intronic	PRIM2	A/G	0.009	-7.926 (1.405)	1.27E-07

Chr, chromosome; SNP, single nucleotide polymorphisms; MAF, minor allele frequency; SE, standard error; K-CHIP, the Korea Biobank Array

Table S2. Genome-wide association study-derived genetic variants in the K-CHIP consortium (Continued)

Chr	BP	SNP	Function	Gene	Alleles	MAF	Beta (SE)	P-value
6p11.2	57328179	rs1218145796	intronic	PRIM2	A/G	0.009	-7.930 (1.406)	1.27E-07
6p11.2	57327372	rs1294551034	intronic	PRIM2	A/T	0.009	-7.930 (1.406)	1.27E-07
6p11.2	57327572	rs1319926816	intronic	PRIM2	G/T	0.009	-7.930 (1.406)	1.27E-07
6p11.2	57330616	rs1334057224	intronic	PRIM2	T/G	0.009	-7.937 (1.407)	1.27E-07
6p11.2	57330720	rs761503009	intronic	PRIM2	A/C	0.009	-7.937 (1.407)	1.27E-07
15q26.1	89197517	rs141786963	intronic	ISG20	C/T	0.022	-5.985 (1.062)	1.28E-07
5q21.3	108575881	rs80194513	ncRNA_intronic	LOC285638	T/C	0.003	-49.798 (8.840)	1.31E-07
4p16.1	11150684	rs375628754	intergenic	CLNK;MIR572	G/T	0.036	-3.913 (0.696)	1.38E-07
3q29	195804208	rs17091588	intronic	TFRC	A/G	0.052	-3.181 (0.566)	1.38E-07
3q29	195798320	rs41301381	exonic	TFRC	G/C	0.052	-3.172 (0.565)	1.44E-07
5p15.33	3278879	rs372457561	intergenic	LINC01377;LINC01019	T/C	0.022	-5.131 (0.915)	1.49E-07
3p24.1	27705156	rs536262664	intergenic	SLC4A7;EOMES	C/T	0.000	-312.603 (56.097)	1.73E-07
13q34	112170827	rs139027045	intergenic	TEX29;LINC02337	C/T	0.010	-7.885 (1.417)	1.80E-07
5p15.2	13361785	rs138135958	intergenic	LINC02220;DNAH5	G/T	0.017	-5.730 (1.030)	1.80E-07
1p33	49684590	rs185947935	intronic	AGBL4	C/T	0.013	-6.262 (1.128)	1.91E-07
3p24.1	29770237	rs79101590	intronic	RBMS3	T/C	0.024	-4.940 (0.891)	1.98E-07
20q13.2	50168870	rs188208200	intronic	NFATC2	C/T	0.010	-7.341 (1.325)	1.99E-07
5q21.1	102568853	rs75498191	intergenic	PPIP5K2;C5orf30	A/G	0.000	-777.631 (140.453)	2.03E-07
1p33	49659599	rs117205909	intronic	AGBL4	C/T	0.013	-6.291 (1.137)	2.07E-07
1p33	49657256	rs184839480	intronic	AGBL4	T/C	0.013	-6.295 (1.138)	2.10E-07
15q26.1	89180627	rs148556246	intronic	ISG20	C/T	0.017	-5.226 (0.946)	2.17E-07
12q23.3	108894982	rs11113949	intergenic	LINC01498;FICD	G/A	0.010	-7.266 (1.316)	2.19E-07

Chr, chromosome; SNP, single nucleotide polymorphisms; MAF, minor allele frequency; SE, standard error; K-CHIP, the Korea Biobank Array

Table S2. Genome-wide association study-derived genetic variants in the K-CHIP consortium (Continued)

Chr	BP	SNP	Function	Gene	Alleles	MAF	Beta (SE)	P-value
12q23.3	108895133	rs11113950	intergenic	LINC01498;FICD	A/C	0.010	-7.266 (1.316)	2.19E-07
12q23.3	108894645	rs11113946	intergenic	LINC01498;FICD	C/A	0.010	-7.266 (1.316)	2.19E-07
12q23.3	108894695	rs11113947	intergenic	LINC01498;FICD	A/G	0.010	-7.266 (1.316)	2.19E-07
12q23.3	108894518	rs11113945	intergenic	LINC01498;FICD	T/C	0.010	-7.265 (1.316)	2.20E-07
12q23.3	108894433	rs12367034	intergenic	LINC01498;FICD	G/A	0.010	-7.265 (1.316)	2.20E-07
12q23.3	108895874	rs11113951	intergenic	LINC01498;FICD	A/G	0.010	-7.265 (1.317)	2.20E-07
7p15.3	21630394	rs143123989	intronic	DNAH11	A/T	0.009	-8.086 (1.466)	2.22E-07
12q23.3	108896797	rs11113953	intergenic	LINC01498;FICD	C/T	0.010	-7.263 (1.317)	2.22E-07
12q23.3	108893730	rs11113944	intergenic	LINC01498;FICD	T/C	0.010	-7.262 (1.316)	2.22E-07
12q23.3	108893870	rs77449249	intergenic	LINC01498;FICD	A/T	0.010	-7.262 (1.316)	2.22E-07
12q23.3	108893479	rs11113940	intergenic	LINC01498;FICD	T/A	0.010	-7.261 (1.316)	2.23E-07
12q23.3	108893524	rs11113941	intergenic	LINC01498;FICD	C/T	0.010	-7.261 (1.316)	2.23E-07
12q23.3	108893600	rs11113942	intergenic	LINC01498;FICD	G/T	0.010	-7.261 (1.316)	2.23E-07
12q23.3	108897590	rs11113956	intergenic	LINC01498;FICD	C/A	0.010	-7.263 (1.317)	2.23E-07
12q23.3	108893722	rs11113943	intergenic	LINC01498;FICD	G/A	0.010	-7.261 (1.316)	2.23E-07
12q23.3	108893341	rs11113939	intergenic	LINC01498;FICD	G/A	0.010	-7.260 (1.316)	2.24E-07
12q23.3	108893177	rs11113936	intergenic	LINC01498;FICD	T/G	0.010	-7.260 (1.316)	2.24E-07
12q23.3	108893204	rs11113937	intergenic	LINC01498;FICD	G/T	0.010	-7.260 (1.316)	2.24E-07
12q23.3	108893248	rs11113938	intergenic	LINC01498;FICD	T/G	0.010	-7.260 (1.316)	2.24E-07
12q23.3	108892840	rs11113935	intergenic	LINC01498;FICD	C/T	0.010	-7.259 (1.317)	2.25E-07
12q23.3	108892921	rs199953283	intergenic	LINC01498;FICD	A/AT	0.010	-7.259 (1.317)	2.25E-07
12q23.3	108892623	rs11113932	intergenic	LINC01498;FICD	A/G	0.010	-7.258 (1.317)	2.26E-07

Chr, chromosome; SNP, single nucleotide polymorphisms; MAF, minor allele frequency; SE, standard error; K-CHIP, the Korea Biobank Array

Table S2. Genome-wide association study-derived genetic variants in the K-CHIP consortium (Continued)

Chr	BP	SNP	Function	Gene	Alleles	MAF	Beta (SE)	P-value
12q23.3	108892405	rs11113930	intergenic	LINC01498;FICD	C/T	0.010	-7.258 (1.317)	2.26E-07
12q23.3	108892085	rs11113927	intergenic	LINC01498;FICD	A/C	0.010	-7.258 (1.317)	2.26E-07
12q23.3	108892316	rs11113928	intergenic	LINC01498;FICD	T/A	0.010	-7.258 (1.317)	2.26E-07
12q23.3	108892317	rs11113929	intergenic	LINC01498;FICD	G/A	0.010	-7.258 (1.317)	2.26E-07
12q23.3	108892094	rs138719215	intergenic	LINC01498;FICD	A/AG	0.010	-7.258 (1.317)	2.26E-07
19q13.43	58196865	rs967336174	intronic	ZNF551	CCT/C	0.010	-7.257 (1.317)	2.27E-07
12q23.3	108891504	rs12368931	intergenic	LINC01498;FICD	A/G	0.010	-7.256 (1.317)	2.28E-07
12q23.3	108891644	rs11113926	intergenic	LINC01498;FICD	G/A	0.010	-7.255 (1.317)	2.28E-07
12q23.3	108891607	rs12372268	intergenic	LINC01498;FICD	C/A	0.010	-7.255 (1.317)	2.28E-07
12q23.3	108891441	rs57853417	intergenic	LINC01498;FICD	A/G	0.010	-7.255 (1.317)	2.29E-07
12q23.3	108898482	rs57507907	intergenic	LINC01498;FICD	T/A	0.010	-7.257 (1.317)	2.29E-07
12q23.3	108890795	rs200436713	intergenic	LINC01498;FICD	T/TA	0.010	-7.252 (1.317)	2.31E-07
12q23.3	108890835	rs11113924	intergenic	LINC01498;FICD	T/A	0.010	-7.251 (1.317)	2.32E-07
15q26.1	89173098	rs150509619	intronic	AEN	A/C	0.018	-5.277 (0.958)	2.32E-07
12q23.3	108890649	rs76473093	intergenic	LINC01498;FICD	G/C	0.010	-7.251 (1.317)	2.32E-07
12q23.3	108890651	rs77258936	intergenic	LINC01498;FICD	T/A	0.010	-7.251 (1.317)	2.32E-07
12q23.3	108898481	rs56999150	intergenic	LINC01498;FICD	C/A	0.011	-7.252 (1.318)	2.35E-07
12q23.3	108898892	rs60988999	intergenic	LINC01498;FICD	C/A	0.010	-7.251 (1.318)	2.36E-07
12q23.3	108890137	rs76822917	intergenic	LINC01498;FICD	A/G	0.010	-7.246 (1.317)	2.36E-07
12q23.3	108890022	rs77049190	intergenic	LINC01498;FICD	T/C	0.010	-7.246 (1.317)	2.36E-07
12q23.3	108890022	rs77049190	intergenic	LINC01498;FICD	T/C	0.010	-7.246 (1.317)	2.36E-07
12q23.3	108889920	rs79379950	intergenic	LINC01498;FICD	A/G	0.010	-7.243 (1.317)	2.39E-07

Chr, chromosome; SNP, single nucleotide polymorphisms; MAF, minor allele frequency; SE, standard error; K-CHIP, the Korea Biobank Array

Table S2. Genome-wide association study-derived genetic variants in the K-CHIP consortium (Continued)

Chr	BP	SNP	Function	Gene	Alleles	MAF	Beta (SE)	P-value
1q43	240811124	rs565547003	intergenic	GREM2;RGS7	A/G	0.012	-6.624 (1.209)	2.62E-07
5p15.2	13341420	rs142344584	intergenic	LINC02220;DNAH5	A/G	0.017	-5.681 (1.037)	2.64E-07
12q24.33	130982880	rs55762517	intronic	RIMBP2	G/A	0.015	-6.217 (1.135)	2.64E-07
13q31.1	79187459	rs1180870680	ncRNA_intronic	OBI1-AS1	T/C	0.026	-4.123 (0.754)	2.74E-07
17p13.2	6091502	rs116979438	intergenic	WSCD1;AIPL1	T/C	0.013	-5.952 (1.091)	2.89E-07
1q43	239701246	rs16838444	intronic	CHRM3	T/G	0.013	-5.952 (1.091)	2.89E-07
12q24.33	130984960	rs60921151	intronic	RIMBP2	G/A	0.015	-6.146 (1.127)	2.97E-07
4q32.3	164687231	rs12505111	intronic	MARCHF1	A/G	0.027	-4.253 (0.782)	3.12E-07
1q21.3	151012068	rs115422057	intronic	BNIPL	G/A	0.010	-7.387 (1.362)	3.36E-07
9q31.1	104627355	rs10989665	intergenic	GRIN3A;LINC00587	A/C	0.045	-3.442 (0.635)	3.40E-07
12q24.33	130985984	rs66756436	intronic	RIMBP2	G/A	0.015	-6.064 (1.123)	3.76E-07
1q21.3	151013099	rs151070704	intronic	BNIPL	C/T	0.010	-7.363 (1.364)	3.78E-07
1q21.3	151014400	rs146753684	intronic	BNIPL	A/G	0.010	-7.363 (1.365)	3.81E-07
1q21.3	151015174	rs117770722	intronic	BNIPL	C/T	0.010	-7.362 (1.365)	3.84E-07
2q36.1	222176106	rs72952909	intergenic	MIR4268;EPHA4	A/G	0.086	-2.375 (0.441)	3.98E-07
8p23.2	4820233	rs146823031	intronic	CSMD1	T/C	0.012	-6.945 (1.290)	4.05E-07
1q21.3	151008914	rs115270318	upstream;downstream	BNIPL;PRUNE1	T/A	0.010	-7.359 (1.368)	4.12E-07
2q36.1	222178867	rs1430246	intergenic	MIR4268;EPHA4	A/G	0.086	-2.367 (0.440)	4.19E-07
2q36.1	222182550	rs16862493	intergenic	MIR4268;EPHA4	C/G	0.086	-2.355 (0.439)	4.44E-07
1q21.3	151007574	rs12070466	UTR3	PRUNE1	G/C	0.011	-7.347 (1.372)	4.58E-07
2q36.1	222190938	rs72952942	intergenic	MIR4268;EPHA4	C/T	0.087	-2.328 (0.435)	4.60E-07
2q36.1	222191045	rs16862504	intergenic	MIR4268;EPHA4	G/A	0.087	-2.328 (0.435)	4.61E-07

Chr, chromosome; SNP, single nucleotide polymorphisms; MAF, minor allele frequency; SE, standard error; K-CHIP, the Korea Biobank Array

Table S2. Genome-wide association study-derived genetic variants in the K-CHIP consortium (Continued)

Chr	BP	SNP	Function	Gene	Alleles	MAF	Beta (SE)	P-value
4q13.3	72010245	rs375674565	intergenic	DCK;SLC4A4	T/G	0.028	-4.228 (0.790)	4.63E-07
4q32.3	164684385	rs13127646	intronic	MARCHF1	G/A	0.027	-4.205 (0.786)	4.65E-07
4q28.1	127035452	rs117610704	intergenic	MIR2054;INTU	A/G	0.010	-7.848 (1.467)	4.70E-07
2q36.1	222186825	rs16862499	intergenic	MIR4268;EPHA4	A/T	0.087	-2.336 (0.437)	4.71E-07
2q36.1	222187315	rs16862501	intergenic	MIR4268;EPHA4	G/A	0.087	-2.335 (0.437)	4.74E-07
2q36.1	222185906	rs72952930	intergenic	MIR4268;EPHA4	C/T	0.087	-2.338 (0.438)	4.85E-07
9q31.1	104638232	rs10989675	intergenic	GRIN3A;LINC00587	G/A	0.044	-3.341 (0.625)	4.85E-07
3q27.2	184792192	rs79424017	intergenic	VPS8;C3orf70	C/T	0.014	-5.823 (1.092)	5.04E-07
9q31.1	104639683	rs12238882	intergenic	GRIN3A;LINC00587	C/T	0.044	-3.325 (0.624)	5.11E-07
11q13.2	68116010	rs112180284	intronic	LRP5	T/C	0.020	-5.100 (0.957)	5.15E-07
3q27.2	184908072	rs183410169	ncRNA_intronic	EHHADH-AS1	T/C	0.008	-8.731 (1.641)	5.33E-07
2q36.1	222191393	rs6748878	intergenic	MIR4268;EPHA4	A/T	0.088	-2.324 (0.438)	5.74E-07
4q21.1	76527346	rs111466342	intronic	CDKL2	G/GT	0.052	-3.092 (0.583)	5.75E-07
9q31.1	104642966	rs9299352	intergenic	GRIN3A;LINC00587	C/T	0.044	-3.289 (0.621)	5.79E-07
1p33	49542012	rs190235218	intronic	AGBL4	C/T	0.012	-6.468 (1.222)	5.95E-07
14q31.1	83551687	rs77734808	intergenic	LINC02301;NONE	G/A	0.016	-6.224 (1.176)	5.98E-07
9q31.1	104645652	rs10989682	intergenic	GRIN3A;LINC00587	G/T	0.044	-3.286 (0.621)	6.13E-07
2q36.1	222184970	rs72952924	intergenic	MIR4268;EPHA4	A/G	0.086	-2.332 (0.441)	6.26E-07
15q26.1	89124390	rs150320390	intergenic	DET1;LINC01586	G/T	0.010	-7.572 (1.434)	6.30E-07
1p34.3	38079825	rs117174295	intronic	RSPO1	C/T	0.026	-4.178 (0.792)	6.43E-07
4q32.3	164691345	rs10013393	intronic	MARCHF1	T/G	0.035	-3.645 (0.691)	6.51E-07
1p33	49501403	rs140975530	intronic	AGBL4	T/G	0.012	-6.463 (1.226)	6.54E-07

Chr, chromosome; SNP, single nucleotide polymorphisms; MAF, minor allele frequency; SE, standard error; K-CHIP, the Korea Biobank Array

Table S2. Genome-wide association study-derived genetic variants in the K-CHIP consortium (Continued)

Chr	BP	SNP	Function	Gene	Alleles	MAF	Beta (SE)	P-value
1q25.3	185746647	rs61314460	intronic	HMCN1	G/A	0.070	-2.555 (0.485)	6.58E-07
3q21.3	126517078	rs138613194	intronic	CHCHD6	C/T	0.007	-11.763 (2.233)	6.66E-07
15q22.2	60473320	rs140501799	intergenic	FOXB1;ANXA2	A/G	0.030	-3.857 (0.732)	6.70E-07
1p34.3	38095804	rs12753021	intronic	RSPO1	C/T	0.027	-4.150 (0.788)	6.79E-07
1p33	49485775	rs146366723	intronic	AGBL4	T/C	0.012	-6.475 (1.231)	6.91E-07
2q36.1	222191389	rs200126617	intergenic	MIR4268;EPHA4	A/T	0.087	-2.322 (0.442)	7.13E-07
1p34.3	38097519	rs71640742	UTR5	RSPO1	A/C	0.026	-4.137 (0.788)	7.18E-07
4q32.3	164704667	rs10009600	intronic	MARCHF1	G/C	0.035	-3.615 (0.689)	7.26E-07
22q12.3	37122086	rs16997246	ncRNA_intronic	LOC105373021	T/C	0.013	-5.769 (1.099)	7.26E-07
4q32.3	164741639	rs28637179	intronic	MARCHF1	G/A	0.035	-3.619 (0.690)	7.29E-07
4q32.3	164744895	rs13103455	intronic	MARCHF1	T/C	0.035	-3.616 (0.689)	7.30E-07
4q32.3	164690172	rs13146066	intronic	MARCHF1	T/C	0.041	-3.437 (0.655)	7.31E-07
4q32.3	164716627	rs10030446	intronic	MARCHF1	C/G	0.035	-3.613 (0.689)	7.44E-07
1q41	221080599	rs74957276	intergenic	HLX;C1orf140	C/T	0.026	-4.132 (0.788)	7.46E-07
1q41	221083429	rs75369607	intergenic	HLX;C1orf140	T/G	0.026	-4.132 (0.788)	7.47E-07
13q14.11	42647779	rs78735820	intronic	DGKH	C/T	0.079	-2.694 (0.514)	7.49E-07
4q32.3	164680214	rs13136324	intronic	MARCHF1	C/T	0.026	-4.143 (0.791)	7.56E-07
4q32.3	164672601	rs13105978	intronic	MARCHF1	T/A	0.026	-4.138 (0.790)	7.68E-07
4q32.3	164672948	rs34162806	intronic	MARCHF1	A/C	0.026	-4.138 (0.790)	7.68E-07
4q32.3	164672979	rs139079372	intronic	MARCHF1	T/TGTGTGA	0.026	-4.138 (0.791)	7.71E-07
4q32.3	164707381	rs4691934	intronic	MARCHF1	G/A	0.035	-3.632 (0.694)	7.78E-07
4q32.3	164714927	rs7695742	intronic	MARCHF1	T/C	0.035	-3.632 (0.695)	7.93E-07

Chr, chromosome; SNP, single nucleotide polymorphisms; MAF, minor allele frequency; SE, standard error; K-CHIP, the Korea Biobank Array

Table S2. Genome-wide association study-derived genetic variants in the K-CHIP consortium (Continued)

Chr	BP	SNP	Function	Gene	Alleles	MAF	Beta (SE)	P-value
13q12.13	27518236	rs11618475	intergenic	GPR12;USP12	A/G	0.017	-5.822 (1.114)	7.97E-07
4q32.3	164670165	rs13101998	intronic	MARCHF1	G/T	0.026	-4.134 (0.791)	7.99E-07
4q32.3	164674901	rs10009742	intronic	MARCHF1	C/T	0.027	-4.128 (0.790)	8.01E-07
4q32.3	164670444	rs1390835129	intronic	MARCHF1	CT/C	0.027	-4.129 (0.790)	8.01E-07
3p24.1	29471087	rs536791351	intronic	RBMS3	A/C	0.018	-5.590 (1.070)	8.04E-07
4q32.3	164670375	rs71600637	intronic	MARCHF1	C/CAT	0.027	-4.127 (0.790)	8.08E-07
4q32.3	164719589	rs7657245	intronic	MARCHF1	G/A	0.035	-3.631 (0.695)	8.09E-07
21q22.12	37599375	rs564113735	intronic	DOP1B	G/C	0.008	-8.378 (1.605)	8.20E-07
18q12.3	39023592	rs80352123	intergenic	LINC01477;KC6	T/A	0.190	-1.950 (0.374)	8.22E-07
4q32.3	164669428	rs12504820	intronic	MARCHF1	C/T	0.026	-4.135 (0.792)	8.26E-07
4q32.3	164668086	rs13139036	intronic	MARCHF1	G/A	0.026	-4.131 (0.792)	8.27E-07
4q32.3	164669328	rs34491561	intronic	MARCHF1	C/A	0.026	-4.135 (0.792)	8.27E-07
4q32.3	164668003	rs17474074	intronic	MARCHF1	G/T	0.026	-4.130 (0.792)	8.32E-07
4q32.3	164694482	rs2201374	intronic	MARCHF1	A/G	0.036	-3.606 (0.691)	8.34E-07
4q32.3	164667565	rs12502759	intronic	MARCHF1	G/A	0.026	-4.129 (0.792)	8.38E-07
4q32.3	164669067	rs12512360	intronic	MARCHF1	A/C	0.026	-4.132 (0.793)	8.45E-07
4q32.3	164666859	rs17577474	intronic	MARCHF1	G/A	0.026	-4.128 (0.792)	8.49E-07
4q32.3	164666401	rs62333006	intronic	MARCHF1	T/C	0.026	-4.127 (0.792)	8.53E-07
4q32.3	164665383	rs6852270	intronic	MARCHF1	C/T	0.027	-4.116 (0.790)	8.55E-07
4p16.1	11229217	rs140390646	intergenic	CLNK;MIR572	G/A	0.040	-3.521 (0.676)	8.55E-07
4q32.3	164665271	rs6847496	intronic	MARCHF1	G/T	0.026	-4.121 (0.791)	8.61E-07
4q32.3	164664668	rs17577453	intronic	MARCHF1	T/C	0.026	-4.120 (0.791)	8.66E-07

Chr, chromosome; SNP, single nucleotide polymorphisms; MAF, minor allele frequency; SE, standard error; K-CHIP, the Korea Biobank Array

Table S2. Genome-wide association study-derived genetic variants in the K-CHIP consortium (Continued)

Chr	BP	SNP	Function	Gene	Alleles	MAF	Beta (SE)	P-value
4q32.3	164664101	rs5012631	intronic	MARCHF1	C/G	0.027	-4.113 (0.790)	8.69E-07
4q32.3	164663804	rs146477132	intronic	MARCHF1	A/AT	0.026	-4.120 (0.791)	8.72E-07
14q22.2	54445014	rs189166361	intergenic	BMP4;CDKN3	G/A	0.033	-3.827 (0.735)	8.80E-07
4q32.3	164663966	rs954805	intronic	MARCHF1	C/T	0.026	-4.122 (0.792)	8.80E-07
5q11.2	56577733	rs140712449	intergenic	GPBP1;ACTBL2	G/C	0.020	-5.826 (1.120)	8.81E-07
4q32.3	164702553	rs28562031	intronic	MARCHF1	C/G	0.035	-3.591 (0.690)	8.88E-07
4q32.3	164660169	rs903300	intronic	MARCHF1	C/T	0.026	-4.115 (0.791)	8.99E-07
4q32.3	164659196	rs9683694	intronic	MARCHF1	G/T	0.026	-4.115 (0.791)	9.00E-07
4q32.3	164659625	rs2131863	intronic	MARCHF1	A/G	0.026	-4.114 (0.791)	9.02E-07
4q35.1	186557519	rs190229637	intronic	SORBS2	G/A	0.017	-4.980 (0.958)	9.02E-07
4q32.3	164656734	rs13142980	intronic	MARCHF1	G/A	0.026	-4.114 (0.791)	9.05E-07
4q32.3	164648499	rs2101769	intronic	MARCHF1	G/A	0.026	-4.114 (0.792)	9.06E-07
4q32.3	164646243	rs3921738	intronic	MARCHF1	G/A	0.026	-4.114 (0.792)	9.08E-07
13q13.1	32777632	rs150859875	intronic	FRY	G/A	0.018	-4.233 (0.815)	9.17E-07
12p13.31	6335550	rs141164457	intronic	CD9	G/A	0.006	-10.581 (2.037)	9.18E-07
4q32.3	164691710	rs2022557	intronic	MARCHF1	A/T	0.034	-3.693 (0.711)	9.25E-07
9q31.3	114650365	rs148002888	intergenic	SHOC1;UGCG	T/C	0.019	-5.027 (0.969)	9.38E-07
4q24	102711373	rs73834300	upstream	BANK1	G/C	0.048	-3.111 (0.600)	9.71E-07
12p12.1	21386509	rs34138322	intronic	SLCO1B1	A/C	0.141	-2.087 (0.403)	9.86E-07
1q21.3	151000257	rs72292296	intronic	PRUNE1	T/C	0.019	-5.813 (1.123)	9.87E-07
3p26.2	3168971	rs193122721	UTR5	TRNT1	G/C	0.014	-5.744 (1.110)	9.91E-07

Chr, chromosome; SNP, single nucleotide polymorphisms; MAF, minor allele frequency; SE, standard error; K-CHIP, the Korea Biobank Array

Table S3. Fine-mapping of the lead SNPs from the results of GWAS

SNP	Position	Function	Alleles	MAF	Beta (SE)	P-value	PIP
rs10009742 on MARCHF1 gene							
rs6847496	164665271	intronic	G/T	0.026	-4.121 (0.791)	8.61E-07	0.16
rs62333006	164666401	intronic	T/C	0.026	-4.127 (0.792)	8.53E-07	0.16
rs17577474	164666859	intronic	G/A	0.026	-4.128 (0.792)	8.49E-07	0.16
rs12502759	164667565	intronic	G/A	0.026	-4.129 (0.792)	8.38E-07	0.16
rs17474074	164668003	intronic	G/T	0.026	-4.130 (0.792)	8.32E-07	0.16
rs13139036	164668086	intronic	G/A	0.026	-4.131 (0.792)	8.27E-07	0.16
rs12512360	164669067	intronic	A/C	0.026	-4.132 (0.793)	8.45E-07	0.16
rs34491561	164669328	intronic	C/A	0.026	-4.135 (0.792)	8.27E-07	0.16
rs12504820	164669428	intronic	C/T	0.026	-4.135 (0.792)	8.26E-07	0.16
rs13101998	164670165	intronic	G/T	0.026	-4.134 (0.791)	7.99E-07	0.16
rs13105978	164672601	intronic	T/A	0.026	-4.138 (0.790)	7.68E-07	0.16
rs34162806	164672948	intronic	A/C	0.026	-4.138 (0.790)	7.68E-07	0.16
rs139079372	164672979	intronic	T/TGTGTGA	0.026	-4.138 (0.791)	7.71E-07	0.16
rs13136324	164680214	intronic	C/T	0.026	-4.143 (0.791)	7.56E-07	0.17
rs71600637	164670375	intronic	C/CAT	0.027	-4.127 (0.790)	8.08E-07	0.18
rs10009742	164674901	intronic	C/T	0.027	-4.128 (0.790)	8.01E-07	0.18
rs6852270	164665383	intronic	C/T	0.027	-4.116 (0.790)	8.55E-07	0.18
rs1390835129	164670444	intronic	CT/C	0.027	-4.129 (0.790)	8.01E-07	0.18
rs13127646	164684385	intronic	G/A	0.027	-4.205 (0.786)	4.65E-07	0.20

GWAS, genome-wide association study; SNP, single nucleotide polymorphism; MAF, minor allele frequency; SE, standard error; PIP, posterior inclusion probability
